# Metagenomic and Metatranscriptomic Analysis of Microbial Community Structure and Gene Expression of Activated Sludge

**DOI:** 10.1371/journal.pone.0038183

**Published:** 2012-05-30

**Authors:** Ke Yu, Tong Zhang

**Affiliations:** Environmental Biotechnology Laboratory, Department of Civil Engineering, The University of Hong Kong, Hong Kong SAR, China; Auburn University, United States of America

## Abstract

The present study applied both metagenomic and metatranscriptomic approaches to characterize microbial structure and gene expression of an activated sludge community from a municipal wastewater treatment plant in Hong Kong. DNA and cDNA were sequenced by Illumina Hi-seq2000 at a depth of 2.4 Gbp. Taxonomic analysis by MG-RAST showed bacteria were dominant in both DNA and cDNA datasets. The taxonomic profile obtained by BLAST against SILVA SSUref database and annotation by MEGAN showed that activated sludge was dominated by *Proteobacteria*, *Actinobacteria*, *Bacteroidetes, Firmicutes* and *Verrucomicrobia* phyla in both DNA and cDNA datasets. Global gene expression annotation based on KEGG metabolism pathway displayed slight disagreement between the DNA and cDNA datasets. Further gene expression annotation focusing on nitrogen removal revealed that denitrification-related genes sequences dominated in both DNA and cDNA datasets, while nitrifying genes were also expressed in relative high levels. Specially, ammonia monooxygenase and hydroxylamine oxidase demonstrated the high cDNA/DNA ratios in the present study, indicating strong nitrification activity. Enzyme subunits gene sequences annotation discovered that subunits of ammonia monooxygenase (*amoA*, *amoB*, *amoC*) and hydroxylamine oxygenase had higher expression levels compared with subunits of the other enzymes genes. Taxonomic profiles of selected enzymes (ammonia monooxygenase and hydroxylamine oxygenase) showed that ammonia-oxidizing bacteria present mainly belonged to *Nitrosomonas* and *Nitrosospira* species and no ammonia-oxidizing *Archaea* sequences were detected in both DNA and cDNA datasets.

## Introduction

Molecular methods based on 16S rRNA genes and function genes, including fluorescence *in situ* hybridization [1], denaturing gradient gel electrophoresis [2], qRT-PCR [3], [4], microarray [5], and proteomics [6], have been conducted to analyze the microbial community structure and gene diversities at various environments. Although those methods are still useful for less diversity communities, those methods may not integrally reflect microbial diversity and couple microbial taxonomy diversity with diversified functions due to low throughput.

High-throughput sequencing methods, such as 454 pyrosequencing and Illumina sequencing technologies, have been recently applied as novel promising methods to investigate the genes and genes expressions levels of microbial community in different habits. DNA based high-throughput sequencing metagenomics have been applied to reveal microbial communities in marine water [7], soil [8], human guts [9], and oral cavities [10]. However, only a few metatranscriptomic studies have been applied to the microbial communities in marine water [11], [12], and soil [8]. No metatranscriptomic work has been conducted on microbial community of activated sludge (AS) from municipal wastewater treatment plant and only a few limited high-throughput sequencing metagenomic studies on AS have been reported [13], [14].

Activated sludge is a widely applied biological process in wastewater treatment plants (WWTP). Similar to soil and sediment, the AS floc is a highly complex microbial system of eukaryotes, bacteria, archaea, and viruses, in which bacteria are dominant and play important roles in removal of organic pollutants and nutrients (nitrogen and phosphorus). To prevent the adverse environmental impacts (including toxicity, oxygen depletion, and algal blooms) caused by ammonia discharge from WWTP, biological nitrification is conducted using AS to oxidize ammonia to nitrite/nitrate via nitrification, and then to nitrogen gas via denitrification. Nitrification is usually the limiting step of the above nitrogen removal process, and catalyzed by two groups of microorganisms, i.e. ammonia-oxidizing microorganism [15], [16] and nitrite-oxidizing bacteria [17]. Although several studies have analyzed ammonia-oxidizing microorganism, nitrite-oxidizing bacteria and denitrifying bacteria using different genes as biomarkers, e.g. ammonia monooxygenase (amo) [14], [18], [19], nitrite reductase subunits (nirK and nirS) [20], etc., a comprehensive study on these genes and their expression levels in AS were never conducted.

In the present study, we studied the microbial community structure and the gene expression levels of an AS from Stanley WWTP in Hong Kong using coupled metatranscriptomic and metagenomic approaches. The aims of the present study were 1) to explore microbial metabolic potential using DNA dataset and transcriptional activity RNA/cDNA dataset in parallel; 2) to characterize the nitrogen metabolism in AS, specifically nitrification and denitrification and the related genes; and 3) to identify the active microorganisms responsible for nitrogen removal in AS.

## Results and Discussion

### Taxon-specific Patterns of rRNA and Protein Coding Sequences

**Figure 1:**
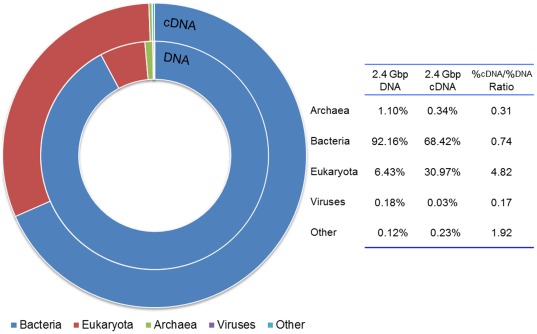
Combined taxonomic domain information of DNA and cDNA datasets. Total DNA sequences and cDNA sequences were assigned to *Bacteria*, *Eukaryota*, *Archaea*, viruses, and other sequences.

After being filtered by MG-RAST based on length and number of ambiguous bases, totally 26,597,304 DNA clean reads (100 bp per read) (∼2.4 Gbp dataset) and 27,999,804 cDNA clean reads (90 bp per read) (∼2.4 Gbp dataset) were used for the analysis. Combined taxonomic results generated based on all the available annotation source databases in MG-RAST are summarized in Figure 1.

Figure 1 showed that bacteria were the dominant domain in both DNA and cDNA datasets, accounting for 92.16% and 68.42% of DNA and cDNA sequences, respectively, while eukaryota comprised approx. 6.43% and 30.97% of total sequences in DNA and cDNA, respectively. Sequences from *Archaea* and viruses only accounted for 1.10% and 0.18% in DNA sequences, and 0.34% and 0.03% in cDNA, respectively.

Comparing metagenomic dataset with metatranscriptomic dataset may reveal the relative activity levels of different populations in a microbial community. In the present study, we defined that the relative activity of a microbial population was its abundance/percentage in cDNA dataset over its abundance/percentage in DNA dataset.

As summarized in Figure 1, the %_cDNA_/%_DNA_ ratios of *Archaea*, *Bacteria*, *Eukaryota*, and viruses were 0.31, 0.74, 4.82, and 0.17, respectively. Although bacterial sequences were the most abundance sequences in both metagenomic and metatranscriptomic datasets, the increase of eukaryotic %_cDNA_/%_DNA_ ratio probably resulted from that the more quick degradation of bacterial mRNA than eukaryotic mRNA.

**Figure 2:**
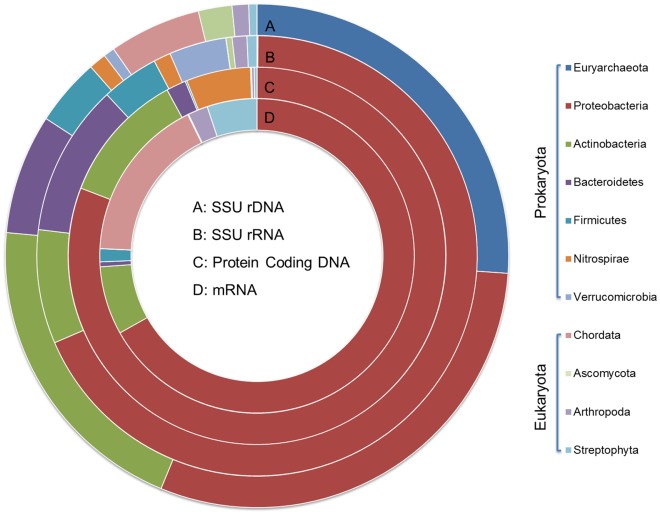
Microbial community composition assessed by taxonomic classification of metagenomic and metatranscriptomic datasets. SSU and LSU rDNA reads, and protein coding DNA reads from metagenomic dataset as well as those reads from metatranscriptomic datasets identified as SSU rRNA, LSU rRNA, and mRNA. Only taxonomic groups that represented >1% of total reads in at least one dataset were included.

For better understanding of the expression level of microorganisms in AS, taxonomic affiliation of rRNA/DNA and protein-coding sequences (mRNA and their genes) were annotated with SSU database or Genbank database of MG-RAST. To simplify the comparison, only taxonomic groups occupying >1% of Best Hit annotated reads in DNA or cDNA dataset were included. Figure 2 showed that the most abundant bacterial populations were *Proteobacteria*, *Actinobacteria*, *Bacteroidetes,* and *Firmicutes*, which accounted for 22.35%, 15.03%, 5.72%, and 3.22% in SSU rDNA reads, respectively, 49.46%, 6.04%, 8.06%, and 3.04% in SSU rRNA reads, 78.47%, 10.93%, 1.64%, and 0.14% in protein coding DNA reads, and 62.82%, 6.62%, 0.47%, and 1.26% of mRNA reads. *Verrucomicrobia* had high abundance in SSU rRNA reads (3.03%), but relatively low abundance in SSU rDNA reads (0.53%), mRNA reads (0.01%), and their corresponding protein coding DNA reads (0.02%). The phylum of nitrite-oxidizing bacteria, *Nitrospirae*, showed high abundance in protein coding DNA reads (5.36%), relative high abundance in SSU rDNA (0.83%), as well as rRNA (0.91%), but low abundance in mRNA (<0.01%). *Euryarchaeota*, an archaeal phylum, also had very high percentage in SSU rDNA (19.38%), but low percentage in SSU rRNA (0.05%), protein coding DNA (0.01%), and mRNA (<0.01%).

High abundance of eukaryotic phyla had been observed in SSU rRNA, SSU rRNA, protein coding DNA, or mRNA sequences. Proportions of mRNA and rRNA in dominant eukaryotic phyla were generally higher than their proportions in protein coding DNA and rDNA. *Chordata* (15.98% in mRNA reads, 0.05% in protein coding DNA reads, 0.21% in SSU rRNA reads and 4.38% in SSU rDNA reads) was the most dominant eukaryote in both DNA and cDNA datasets. High abundance of *Chordata* mRNA was mostly comprised of *Mammalia*, *Aves*, and *Actinopterygii* (70.65%, 24.52%, and 4.99% in total *Chordata* sequences, respectively). Although living *Chordata* cell may reach aerobic tank by influent, it is interesting that high abundance of *Mammalia*, *Aves*, and *Actinopterygii* mRNA survive in aerobic tank. *Streptophyta* also has high gene expression activity in mRNA (4.82%), but relative low percentages in protein coding DNA reads (0.23%), SSU rRNA (0.53%) and SSU rDNA reads (0.39%), suggesting that *Streptophyta* in AS may adapt to a low light condition, due to Stanley WWTP is a treatment plant inside an artificial cave with low light condition. The other phyla, such as *Arthropoda* and *Ascomycota*, also had relatively high abundance in mRNA (1.92%) and SSU rDNA (1.60*%)*, respectively.

Table 1 showed that bacteria had much lower expression activity level in terms of protein coding genes than eukaryotes, although their rRNA expression levels were generally higher than eukaryotes. Consistent results were observed in high abundant bacterial and eukaryotic phyla. The %_SSU rRNA_/%_SSU rDNA_ ratios of the top four abundant eukaryotes ranged from less than 0.01 to 1.36, while %_SSU rRNA_/%_SSU rDNA_ ratios ranged from 0.40 to 5.72 for the top six abundant bacterial phyla. Except for *Firmicutes*, which had a similar %_mRNA_/%_protein coding DNA_ ratio to *Arthropoda*, dominant bacterial %_mRNA_/%_protein coding DNA_ were dramatically lower than those of eukaryotes. The extremely low expression ratio of rRNA and high expression level of mRNA in the most abundance eukaryotic phylum (*Chordata*), as well as low mRNA expression ratio but relative high ratio of rRNA expression in the most abundant bacterial phyla may largely be responsible for the phenomenon. These results also indicated that bacteria possibly expressed higher levels of rRNA, but their mRNA degraded faster than eukaryotic mRNA. High expression levels of rRNA and fast mRNA degradation possibly help bacteria to fast response to changing environment.

**Table 1:** Microbial community composition assessed by taxonomic classification of metagenomic and metatranscriptomic sequence reads.

	SSU rRNA (%)/SSU rDNA (%)	mRNA (%)/protein coding DNA (%)
***Archaea***	<0.01	<0.01
***Bacteria***	1.47	0.74
***Eukaryota***	0.66	23.03
**Virus**	0.67	3.83
***Euryarchaeota***	<0.01	<0.01
***Proteobacteria***	2.21	0.80
***Actinobacteria***	0.40	0.61
***Bacteroidetes***	1.41	0.29
***Firmicutes***	0.93	9.00
***Nitrospirae***	1.10	<0.01
***Verrucomicrobia***	5.72	0.50
***Chordata***	<0.01	319.60
***Ascomycota***	0.20	8.00
***Arthropoda***	0.99	8.73
***Streptophyta***	1.36	20.96

Results showed that bacteria have much lower community expression efficiency of protein coding genes, compared with eukaryote. However, their rRNA expression levels were generally higher than eukaryote.

Further analysis was also performed to calculate the proportions of *Archaea*, *Bacteria*, and *Eukaryota* between annotated SSU and LSU sequences. Best Hit results by MG-RAST using SSU and LSU databases, showed disagreement of the proportions of *Archaea*, *Bacteria*, and *Eukaryota*, which probably resulted from different coverage of SSU and LSU databases. Archaeal, bacterial, or eukaryotic rDNA sequences accounted for 17.6%, 52.1% and 10.9% of annotated SSU sequences, and 0.02%, 86.1%, and 9.6% of annotated LSU sequences, respectively (Table 2). 18.4% of annotated SSU rDNA sequences could not be assigned to *Archaea*, *Bacteria*, or *Eukaryota*. In cDNA dataset, the proportion of *Eukaryota* varied from 6.5% (SSU) to 19.4% (LSU). However, the percentages of both *Archaea* and *Bacteria* in cDNA dataset were at similar levels using different annotation sources. In addition, the percentages of unassigned reads in DNA dataset were almost at the same level, while they varied in cDNA dataset from 18.4% (SSU) to 6.2% (LSU).

**Table 2:** Percentage (%) of *Archaea*, *Bacteria*, and *Eukaryota* in annotated SSU and LSU sequences.

	DNA		cDNA	
	SSU (%)	LSU (%)	SSU (%)	LSU (%)
*Archaea*	17.6	0.02	0.05	0.01
*Bacteria*	52.1	86.1	75	74.4
*Eukaryota*	10.9	9.6	6.5	19.4
Unassigned	0.01	0.02	18.4	6.2

Disagreement was found in DNA dataset between annotated SSU and LSU sequences. As well, the percentages of *Eukaryota* in annotated SSU and LSU sequences varied while *Archaea* and *Bacteria* kept at similar levels.

### Taxonomic Classification

BLASTN results showed that portion of SSU coding genes (16S/18S rDNA) and LSU coding genes (23S/28S rDNA) in total DNA sequences (∼2.4 Gbp DNA dataset) was roughly 0.3%; while assigned SSU and LSU rRNA sequences accounted for ∼71.2% in total cDNA sequences, which is similar to the SSU and LSU rRNA portion in soil (74.8%) [8], but higher than marine environment (37.1%∼58.1%) [12].

BLAST results, which were annotated by MEGAN by lowest common ancestor algorithm, showed that archaeal, bacterial, or eukaryotic rDNA sequences accounted for 17.1%, 63.1% and 15.8% of annotated SSU sequences, which was in agreement with those results using Best Hit of MG-RAST. Because there was roughly 71.2% of total RNA sequences came from SSU and LSU rRNA, for a fair comparison with DNA dataset, total 117855 cDNA sequences were randomly picked out from the total 2.4 Gbp cDNA for SSU taxonomic annotation. *Bacteria* and *Eukaryota* took 73.2% and 18.9% in total assigned SSU rRNA reads, respectively, while there was almost no *Archaea* SSU rRNA reads. These findings showed slight difference between activated sludge and soil microbial community, in which 87.2%, 1.5%, and 10.3% of SSU rRNA belonged to *Bacteria*, *Archaea*, and *Eukaryota*, respectively [12]. The different between soil and AS, such as low oxygen concentration in soil but high oxygen concentration in AS, may be responsible for the different. Comparing to archaeal SSU rDNA accounting for 17.1% in SSU rDNA, low level of archaeal SSU rRNA indicated their low activity in the AS system, which was very reasonable due to the aerobic condition.

MEGAN analysis at the phylum level (Figure S1) showed similar results with MG-RAST Best Hit results. Most of the bacterial phyla, all the archaeal phyla, and many of the eukaryotic phyla (*Actinobacteria*, *Firmicutes*, *Planctomycetes*, *Euryarchaeota*, *Foraminifera*, etc.) had %_SSU rRNA_/%_ SSU rDNA_ ratios lower than 1. Meanwhile, the %_SSU rRNA_/%_ SSU rDNA_ ratios of many bacteria phyla (e.g. *Proteobacteria, Acidobacteria, Bacteroidetes, Verrucomicrobia,* etc.), almost of all fungi (*Chytridiomycota, Ascomycota*, and *Basidiomycota*), and some phyla of the metazoan group (*Ciliophora, Nematoda* and *Rotifera*) were higher than 1. These findings indicated the different SSU expression abilities among phyla and, possibly, their relative activities in the activated sludge. What should be pointed out that because of the limitation of metagenomic information, which based on DNA from both live and dead cells, taxonomic analysis based on metatranscriptome reflected live/active microbial community more accurately than metagenomic data.

*Proteobacteria* were the most abundant phyla in AS, accounting for 37.5% and 56.1% of annotated bacterial SSU rDNA and rRNA sequences, respectively, followed by *Bacteroidetes, Verrucomicrobia*, and *Actinobacteria*, in agreement with our results using V4 16S rRNA gene pyrotags obtained using 454 pyrosequencing [14]. Similar results were also found in a few previous studies on activated sludge using microarray [5] and cloning [21], as well as the analysis results of bacteria communities in sewage influent [22], in which *Proteobacteria* was the most dominant community.

### Global Gene Expression Analysis

Although metatranscriptomes might be expected to have lower microbial richness compared with metagenomes as expression is limited to some populations in the whole microbial community at a given time, for some cases, they may have higher coverage than metagenomes at the same size of a sequence library [7], [23]. 2.4 Gbp DNA and cDNA datasets were used to analysis. The mapping results (Figure S2) showed slight different annotation coverage of the KEGG global metabolism pathway between the two datasets. The blue regions showed those metabolisms only covered by DNA sequences, indicating the expression of these metabolism pathways may not be detectable or activated in the sample at the applied sequencing depth. The red regions only covered by cDNA dataset indicated those metabolisms expression could be detected at the applied sequencing depth in spite of its lower abundance in metagenomic dataset. The purple regions in the metabolic map suggest those pathways detected in both DNA and cDNA datasets.

**Figure 3:**
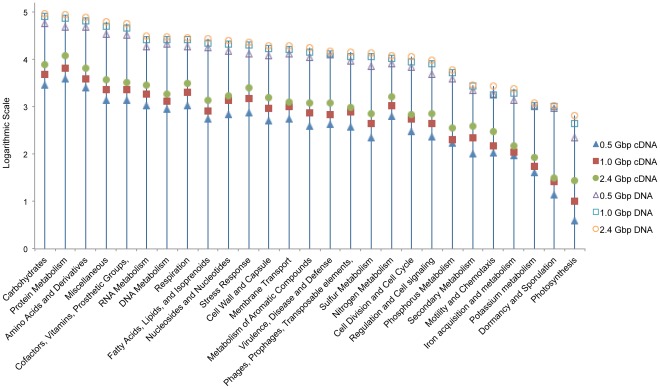
Gene expression classification based on automated SEED subsystem in MG-RAST. Total six datasets were annotated by Level 1 subsystems.

To supplement the information of hit reads abundance in KEGG mapper, the datasets were further annotated with SEED Level1 Subsystems of MG-RAST. Figure 3 shows sequences abundance of 27 basic metabolic categories in six datasets. 495036, 681532, 807124, sequences were annotated from 0.5 Gbp DNA, 1.0 Gbp DNA, and 2.4 Gbp DNA datasets, respectively, while 24425, 41846, and 67014 sequences were annotated from 0.5 Gbp cDNA, 1.0 Gbp cDNA, and 2.4 Gbp cDNA datasets, respectively. Results showed that at the same sequencing depth, abundance of annotated genes in cDNA datasets were generally an order of magnitude lower than those in DNA datasets. Deeper sequencing significantly increased the numbers of the annotated sequences in all groups for both DNA and cDNA datasets. Similar to the findings about soil [8], and marine microbial communities [7], protein metabolism, carbohydrates, amino acids and derivatives, as well as miscellaneous (e.g. translation elongation factor LepA, DNA-directed RNA polymerase alpha subunit, etc.) were the four most abundant categories in the global metabolism of AS microbial communities, suggesting the dominant roles of these categories in microorganisms. Although the total hit numbers of sequences were different between DNA (807124 reads in total 2.4 Gbp DNA data) and cDNA (67014 reads in total 2.4 Gbp cDNA data), the percentages of most categories were similar in DNA and cDNA datasets, for instance, carbohydrates (11.81% and 11.57% in 2.4 Gbp DNA and 2.4 Gbp cDNA, respectively), amino acids and derivatives (9.78% and 9.72% in 2.4 Gbp DNA and 2.4 Gbp cDNA, respectively). However, percentages of protein metabolism, which was the most abundant process found in both datasets, showed a difference between 2.4 Gbp DNA (10.87%) and 2.4 Gbp cDNA (18.10%), suggesting their critical roles and high expression activity in AS. These results were quite lower than that reported in a study on soil microbial community [8], where sequences annotated to protein metabolism accounted for more than 40% in soil microbial community, but slightly higher than that of marine microbial communities, which was around 8% in both DNA and cDNA datasets [7].

Another category which is important in biological nitrogen removal of wastewater, nitrogen metabolism, had hit numbers of 8255, 10062 and 12138 in 0.5 Gbp, 1.0 Gbp, and 2.4 Gbp DNA datasets, and 624, 1042, and 1603 in 0.5 Gbp, 1.0 Gbp, and 2.4 Gbp cDNA datasets, respectively. Accounting for ∼1.5% in 2.4 Gbp DNA dataset and ∼2.4% in 2.4 Gbp cDNA dataset, the portion of nitrogen metabolism related sequences in the total sequences were very similar to the portion in marine microbial communities (around 1.2% in both DNA and cDNA datasets) [7] and soil microbial community (around 2.1% in cDNA dataset) [8].

In addition, extremely low abundant categories, e.g. dormancy and sporulation, and photosynthesis processes, were observed in the present study, possibly due to that Stanley WWTP located in a cave and had average temperature above 20°C, even in winter time.

### Nitrogen Metabolism Analysis

**Figure 4:**
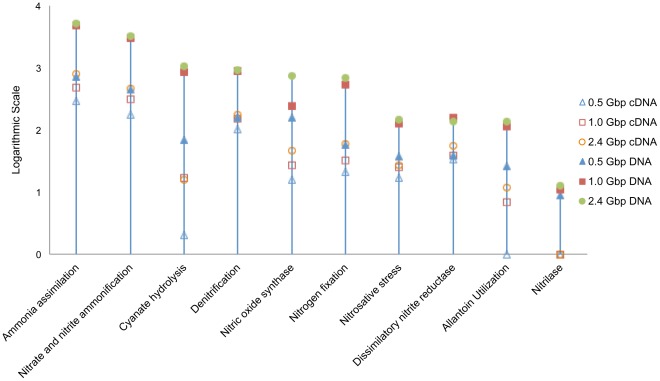
Nitrogen metabolism classification analysis based on level 2 SEED subsystems. Six datasets have been analyzed.

Sequences associated with nitrogen metabolism, especially nitrification, denitrification, ammonification, and nitrogen fixation processes were analyzed in the present study because of their critical roles in nitrogen removal in WWTPs. For the nitrogen metabolism classification analysis, Level 2 SEED Subsystems were used for annotation using MG-RAST. Ammonia assimilation- and nitrite/nitrate ammonification-related genes had the highest hit numbers in both DNA and cDNA datasets, followed by denitrification, nitric oxide synthesis, and nitrogen fixation processes (Figure 4).

**Figure 5:**
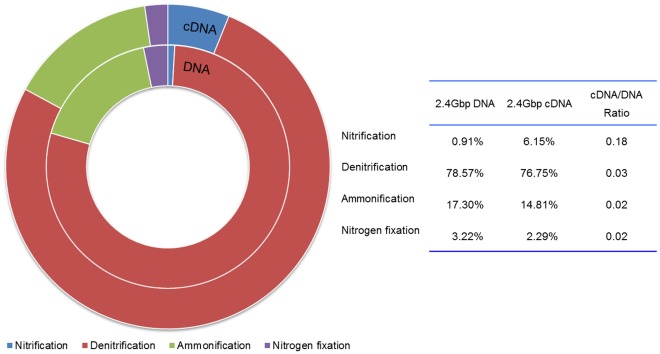
Functional genes and their expression levels in nitrification, denitrification, ammonification, and nitrogen fixation processes.

Sequences associated with these four processes, i.e. ammonification, nitrification, denitrification, and nitrogen fixation, were extracted according to BLAT results for further BLAST against NCBI-nr database and then mapped with MEGAN KEGG analyzer based on the BLAST results. Relevant genes and genes expression levels of nitrification, denitrification, ammonification, and nitrogen fixation processes were displayed in Figure 4 based on the six datasets mentioned in Section 2. Overall, at all three sequencing depths, denitrification had the highest hits among the four processes, followed by ammonification, nitrogen fixation and nitrification (Figure 5). For 2.4 Gbp DNA and cDNA datasets, denitrification accounted for 78.57% and 76.75% of the sum of the four processes, respectively (Figure 5), while ammonification only accounted for 17.30% and 14.81%, nitrogen fixation 3.22% and 2.29%, and nitrification 0.91% and 6.15% of the sum of the four processes, respectively. The results suggested that, both denitrification coding genes sequences and their expression activities were dominant among these four processes. Although the present results showed a small proportion of nitrification-related gene existed in AS microbial community, these genes displayed vigorously expression activity in AS, which expression ratio (cDNA/DNA ratio) was much higher than denitrification, ammonification, and nitrogen fixation.

**Figure 6:**
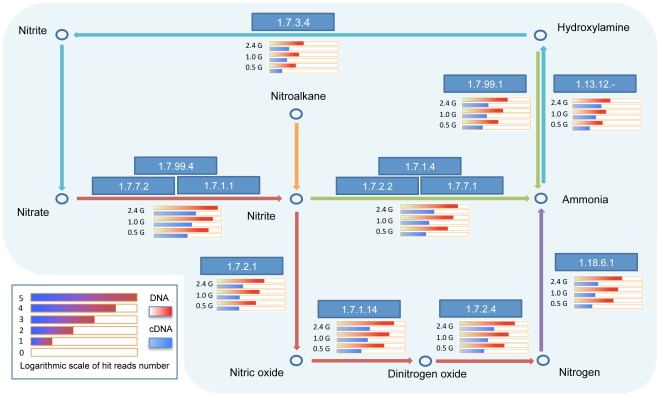
Community abundance of enzymes sequences in ammonification, denitrification, nitrification, and nitrogen fixation (ammonia monooxygenase, hydroxylamine reductase, hydroxylamine oxidase, nitrate reductase, nitric oxide reductase, nitrite reductase, nitrous oxide reductase, and nitrogenase).

Abundance of DNA sequences related to ammonification, denitrification, nitrification, and nitrogen fixation (ammonia monooxygenase (*amo*), hydroxylamine reductase (*har*), hydroxylamine oxidase (*hao*), nitrate reductase (*nar*), nitric oxide reductase (*nor*), nitrite reductase (*nir*), nitrous oxide reductase (*nos*), and nitrogenase (*nif*) were also calculated and shown in Figure 6.

In 2.4 Gbp DNA dataset, coding genes of denitrification enzymes (red line in Figure 6), such as *nir* (including enzymes EC 1.7.1.1, EC 1.7.7.2, and mainly EC 1.7.99.4) coding gene sequences (with total hit number of 44748), *nor* (EC 1.7.2.4) coding gene sequences (12691 hits), and *nos* (EC 1.7.99.6) coding gene sequences (9467 hits) had high abundance, while *nir* (EC 1.7.2.1) (forming NO) coding gene sequences only had 2610 hits. High abundance of ammonification enzymes coding gene sequences were also found, including *nir* (EC 1.7.1.4, EC 1.7.7.1, and EC 1.7.2.2) coding gene sequences (13400 hits) and *har* (EC 1.7.99.1) coding gene sequences (1905 hits). In nitrogen fixation process, 2855 sequences were annotated with *nif* (EC 1.18.6.1) coding gene. In all the four processes, abundance of nitrification enzymes coding gene sequences was the lowest. *amo* (EC 1.13.12.-) and *hao* (EC 1.7.3.4) coding gene sequences only got 518 and 286 hits, respectively. The hit number of *nar* coding gene sequences was ∼86 times of *amo* coding gene sequences and ∼156 times of *hao* coding gene sequences, while abundance of *nos* (EC 1.7.2.4) coding gene sequences were ∼18 and ∼44 times of *amo* and *hao* coding genes sequences, respectively; and *nir* coding sequences were ∼5 and ∼12 times of *amo* and *hao* coding genes sequences, respectively.

The abundance of annotated cDNA sequences in all the four processes were only 1/60 to 1/10 of those in DNA dataset of the same depth (Figure 6). In 2.4 Gbp cDNA dataset, the most abundant enzyme mRNA sequence was *nar* (EC 1.7.1.1, EC 1.7.72, and EC 1.7.99.4, total 1104 hits) (Figure 6), followed by *nos* (EC 1.7.2.4) mRNA sequences (308 hits), *nir* (EC 1.7.1.4, EC 1.7.7.1, and EC 1.7.2.2) mRNA sequences (total 264 hits), *nor* (EC 1.7.2.4) mRNA sequences (228 hits), *amo* (EC 1.13.12.-) mRNA sequences (117 hits), *har* (EC 1.7.99.1) mRNA sequences (78 hits), *nir* (EC 1.7.2.4) mRNA sequences (76 hits), *nif* (EC 1.18.16.1) mRNA sequences (53 hits), and *hao* (EC 1.7.3.4) mRNA sequences (25 hits).

Nitrifying enzymes coding genes were highly expressed in AS. The cDNA/DNA ratio of *amo* was 0.23, 6.9 to 12.6 times higher than those of denitrification enzymes sequences, which ranged from ∼0.02 (*nor*) to ∼0.03 (*nos*). Hydroxylamine oxidase, as well, had a cDNA/DNA ratio of ∼0.09. These ratios suggest that the two nitrification enzymes, especially *amo*, have much higher expression activities in AS. Ammonia monooxygenase had also been found at high expression levels in other ecosystems. Gifford et al. [23] reported that N cycle expression was dominated *amo* transcripts in marine *Alphaproteobacteria*, *Gammaproteobacteria*, and *Bacteroidetes* genomes, when the concentrations of NH_4_^+^ and NO_x_ were about 2.5 µM, and 1.1 µM, respectively. One possible reason responsible for the higher expression of *amo* in AS could be the high concentration of ammonia in the sewage. A previous study based on reverse-transcription PCR found that higher ammonia concentration increase expression of *amoA*-like gene in bulk soil samples [24]. Table S1 showed that the monthly average NH_3_-N concentration of Stanley WWTP influent and effluent were 1.3 mM and 0.02 mM, respectively, from the year 2010 to 2011. Considering the ammonia concentration (average concentration >20 µM) in aerobic tank in Stanley WWTP, which was much higher than that in the ocean [23], high expression levels of *amo* was expected.

**Figure 7:**
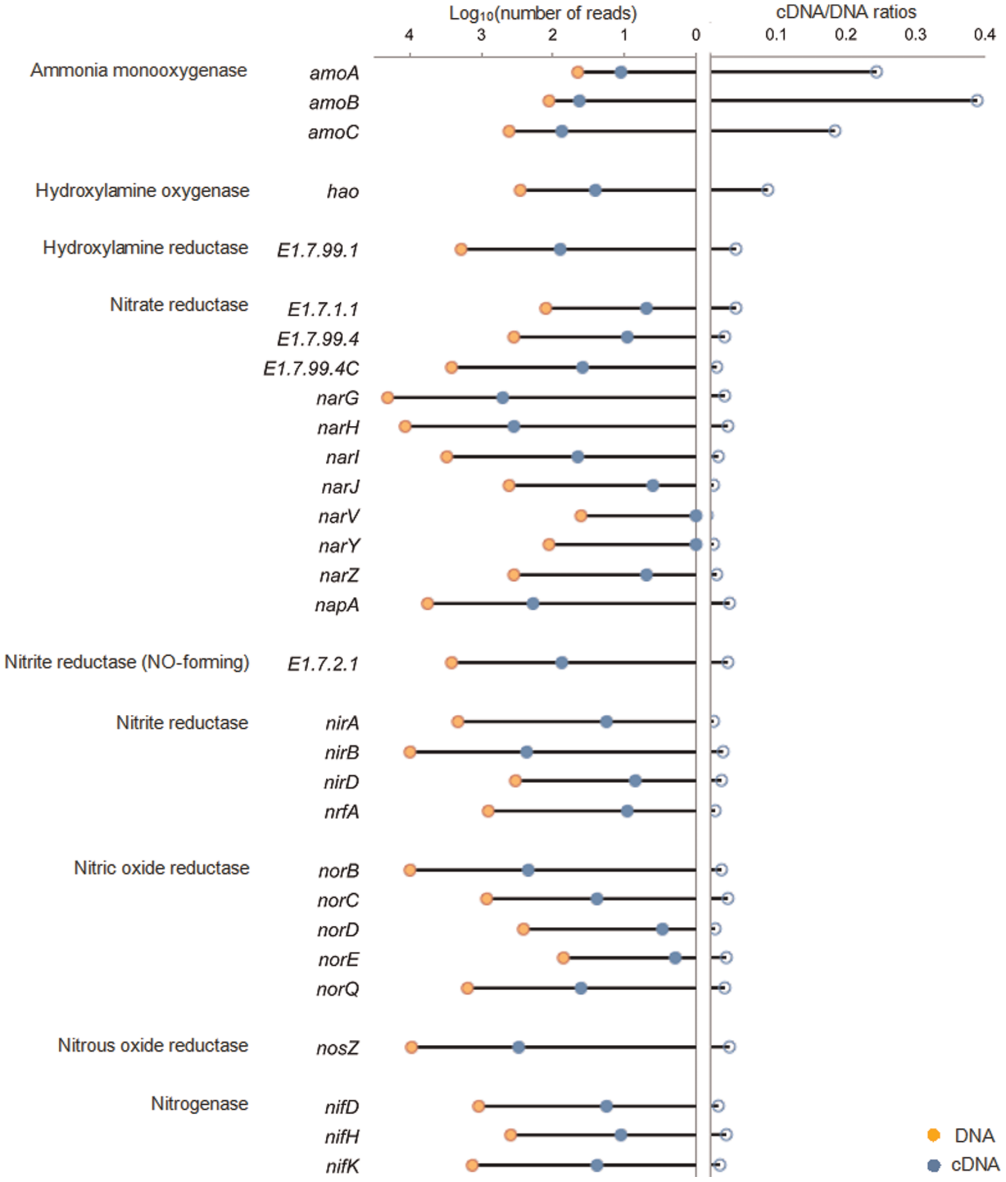
Community abundance of enzymes subunits associated with ammonification, denitrification, nitrification, and nitrogen fixation.

Consistent with above results, most subunits of *nar*, *nir*, *nor*, and *nos* coding genes had very high abundance in DNA dataset (e.g. *narG*, 20344 hits; *narH*, 11713 hits; *nirB*, 10153 hits; *norB*, 9985 hits; *nosZ*, 9467 hits), but relatively low in cDNA dataset (e.g. *narG*, 502 hits; *narH*, 358 hits; *nirB*, 230 hits; *norB*, 218 hits; *nosZ*, 308 hits). Consequently, the ratios of cDNA/DNA of these subunits were lower than 0.04 in the applied sequencing depth (2.4 Gbp) (Figure 7). In comparison, *amoA*, *amoB*, and *amoC* (three subunits of *amo*) have very high cDNA/DNA ratios, which were 0.24, 0.38, and 0.18, respectively, confirming the subunits of *amo* have much higher expression activities as above discussed. This is consistent with the conditions at the sampling point, which was the aeration tank with high DO level (2.5∼3.0 mg L^−1^) favoring nitrification but not denitrification. What should be pointed out is that even though low abundance of nitrification-related genes were observed in DNA dataset, which also suggested that nitrifying microorganisms possibly comprised only a little proportion in AS microbial community, these nitrifying microorganisms with high expression activity performed efficiently in nitrification in AS, reducing ammonia concentration from 1∼1.6 mM in influent to 7∼43 µM in effluent.

### Taxonomic Analysis of the Five Enzymes Coding Sequences and mRNA Sequences

**Figure 8:**
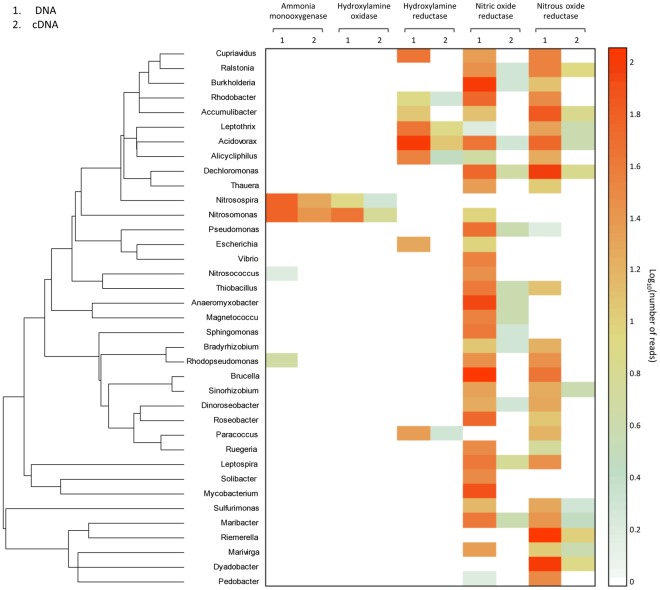
Functional microorganisms in denitrification, nitrogen fixation, as well as nitrification processes. 37 genera of bacteria, containing sequences at least one of the coding sequences of ammonia monooxygenase, hydroxylamine reductase, hydroxylamine oxidase, nitric oxide reductase, and nitrous oxide reductase, were displayed with genus information.

MG-RAST BLAT results containing taxonomic information of hit sequences were further applied to identify species performing nitrification and denitrification. For the nitrification process, the numbers of hit sequences of five enzymes cDNA/DNA sequences and their correlating species were summarized in Figure 8. There were 37 genera of bacteria, containing sequences of at least one of the five enzymes, i.e. ammonia monooxygenase, hydroxylamine reductase, hydroxylamine oxidase, nitric oxide reductase, and nitrous oxide reductase.

Results from 2.4 Gbp DNA dataset showed *Nitrosomonas* (63 hits) and *Nitrosospira* (63 hits) had the highest abundance of ammonia monooxygenase. *Methylocystis* and *Methylosinus* have also been annotated with low abundance of the methanol/ammonia monooxygenase (7 hits and 10 hits, respectively). These two genera have been reported that contain methanol/ammonia monooxygenase in a previous study [25]. The other annotated *amo* sequences mainly came from several groups of uncultured bacteria, e.g. uncultured ammonia-oxidizing beta proteobacterium (total 32 hits), uncultured ammonia-oxidizing bacterium (total 27 hits), and uncultured bacterium (total 22 hits). For another nitrification enzyme, *hao*, the corresponding highest abundant bacteria in the 2.4 Gbp DNA dataset was *Nitrosomonas* (47 hits), followed by *Nitrosospira* (8 hits), *Methylocystis* (4 hits), and *Anaeromyxobacter* (2 hits). The other sequences hit *hao* from uncultured bacterium (57 hits) suggested the potential uncultured bacteria in nitrification. For hydroxylamine reductase, most of the hit sequences came from *Acidovorax* (104 hits), *Cupriavidus* (48 hits), *Leptothrix* (46 hits), *Alicycliphilus* (38 hits), *Paracoccus* (24 hits), *Escherichia* (20 hits), and so on. For nitrous oxide reductase, high abundant annotated bacteria included *Riemerella* (118 hits), *Dyadobacter* (107 hits), *Dechloromonas* (100 hits), *Candidatus accumulibacter* (71 hits), *Acidovorax* (58 hits), and etc. However, the datasets also contained 612 hits of nitrous oxide reductase from uncultured bacterium, indicating the roles of some uncultured bacteria in denitrification.

In comparison, hit reads from cDNA dataset were relative lower. Comparing with DNA dataset, cDNA contained only very little sequences of *amo*, which were assigned to two well-descripted genera, i.e. *Nitrosomonas* (27 hits) and *Nitrosospira* (20 hits), and a group of uncultured ammonia-oxidizing beta proteobacterium (20 hits). Hydroxylamine oxidase was only been annotated to *Nitrosomonas* (6 hits), *Nitrosospira* (2 hits) and a group of uncultured bacterium (8 hits). Hydroxylamine reductase also only had relatively low abundance in *Acidovorax* (12 hits), *Leptothrix* (8 hits), *Vibrio* (5 hits), *Lutiella* (4 hits), *Alicycliphilus* (3 hits). For nitrous oxide reductase, the highest abundant annotated bacteria was *Riemerella* (10 hits), followed by *Ralstonia* (9 hits), *Dyadobacter* (8 hits), *Dechloromonas* (7 hits), *Candidatus accumulibacter* (7 hits), and etc. For nitric oxide reductase, only two genera (*Leptospira* and *Dechloromonas*) with hit sequences were higher than 5.

Further taxonomy-related gene expression activity analysis suggested that *Nitrosomonas* and *Nitrosospira* vigorously expressed ammonia monooxygenase in AS. The present results showed that ammonia monooxygenase relative expression ratios (mRNA/DNA coding sequence) in *Nitrosomonas* and *Nitrosospira* were 0.43 and 0.32, respectively. Except for *Marivirga*, a genus expressed nitrous oxide reductase in ratio of about 0.36, the two genera had the highest expression activity, indicating *Nitrosomonas* and *Nitrosospira* functioned as high efficient *amo* expressers and played dominant roles in nitrification of AS. The present taxonomic analysis results based on SSU showed *Nitrosomonas* and *Nitrosospira* were accounted for 0.11% and 0.02% in DNA dataset, but 0.50% and 0.18% in cDNA dataset. %_SSU rRNA_/%_SSU rDNA_ ratios of these genera were 4.5 and 9, respectively, while the average bacterial %_SSU rRNA_/%_SSU rDNA_ ratio was 1.47 (Table 1). These results suggested *Nitrosomonas* and *Nitrosospira* have high transcription activity in spite of their low abundance in AS. In addition, another genus of nitrifying bacteria, *Nitrosovibrio*, was also detected in both DNA and cDNA datasets from SSU based taxonomic analysis (0.02% and 0.11%, respectively). However, none of *amo* or *hao* was annotated with *Nitrosovibrio*, possibly due to *Nitrosovibrio* have low abundance *amo* or *hao* gene or expressed low abundance of *amo* or *hao* in AS. The insufficient sequencing depth in cDNA dataset may be also responsible for this phenomenon. The present SSU results also showed *Methylocystis* accounted for 0.57% and 0.51% in total SSU rDNA and rRNA, respectively, which %_SSU rRNA_/%_SSU rDNA_ ratio was lower than average bacterial %_SSU rRNA_/%_SSU rDNA_ ratio. But none of SSU rDNA and rRNA sequence was annotated with *Methylosinus*. Combined with the *amo* annotation data mentioned above, none of *amo* mRNA sequences was assigned to *Methylocystis* and *Methylosinus* probably resulted from their relatively low expression activity and insufficient sequencing depth. In accordance with our recently published papers [14], [19], sequences of ammonia-oxidizing *Archaea* were not detected in activated sludge of the present study, implying their minor role in nitrification of sewage.

### Summary

To our knowledge, the present study is the first research on microbial community structure and gene expression of AS by applying combined metagenomic and metatranscriptomic approaches. The present study is also the first study to reveal the abundance and expression levels of genes involved in nitrification, denitrification, ammonification and nitrogen fixation processes in AS sample.

In conclusion, this study presented both metagenomic and metatranscriptomic analysis of an activated sludge sample from a municipal wastewater treatment plant of Hong Kong to characterize microbial community structure and gene expression level. Metagenomic and metatranscriptomic data were analyzed by BLASTN against SILVA SSUref and LSUref databases for taxonomic annotation and MG-RAST sever for functional annotation simultaneously. Results revealed the microbial community structure of AS based on SSU rRNA gene and rRNA (cDNA) sequences; explored nitrogen metabolism by three sequencing depths; analyzed specific metabolism pathway, such as ammonification, denitrification, nitrification, and nitrogen fixation processes in AS; and investigated the taxonomic profile of specific enzymes gene sequences involved in these processes.

In summary, SSU rDNA/rRNA results showed different profiles of microbial community using DNA and cDNA datasets. The percentage of bacterial sequences in AS shifted from 92.2% (DNA) to 68.2% (cDNA). Global gene expression annotation based on KEGG metabolism pathway displayed slight disagreement between the DNA and cDNA datasets. Level 2 SEED subsystems classification showed that ammonia assimilation, nitrite/nitrate ammonification were the highest abundant processes in nitrogen metabolism in both DNA and cDNA datasets, followed by denitrification, nitric oxide synthesis, and nitrogen fixation processes. Nitrification related genes, comprising only ∼1% in DNA dataset but ∼6.8% in cDNA dataset, suggested its high relative expression level in AS microbial community. Enzyme subunits gene sequences annotation discovered that subunits of ammonia monooxygenase (*amoA*, *amoB*, *amoC*) and hydroxylamine oxygenase had higher expression levels compared with subunits of the other enzymes gene sequences. Further characterization of taxonomic profile of five selected enzymes showed that nitrifying bacteria present were affiliated with *Nitrosomonas* and *Nitrosospira*, and, no ammonia oxidizing *Archaea* was in this AS sample at the applied sequencing depth.

## Materials and Methods

### Activated Sludge Sampling

Activated sludge sample was collected from the aeration tank of a local wastewater treatment plant (Stanley) in Hong Kong (N: 114.22, E: 22.21) using RNase and DNase-free tubes on Feb. 15^th^, 2011, immediately frozen in a liquid nitrogen container and transported to the laboratory for future treatment. No specific permits were required for the described field studies. We

- confirm that the location is not privately-owned or protected in any way;
- confirm that the field studies did not involve endangered or protected species.

### DNA Extraction

DNA extraction was conducted within 48 hours after sampling. Briefly, 10 mL samples were centrifuged at 10,000×g for 10 min at 4°C. Two hundred milligrams of the pellet of sample were collected in duplicate for two DNA independent extraction reactions using the FastDNA® SPIN Kit for Soil (Q-Biogene, CA) following the instruction of the manufacturer.

### Total RNA Isolation and cDNA Synthesis

Total RNA isolation was carried out immediately after sampling with the PowerSoil Total RNA Isolation Kit (MO-BIO Laboratories, Inc., CA). Briefly, the frozen sludge sample was quickly thawed, and centrifuged immediately at 13,000×g for 2 min at 4°C. The pellet was collected in duplicate for two independent total RNA isolation reactions following the instruction of the manufacturer. RNA qualification was carried out by electrophoresis (Figure S3). The extracted RNA was dissolved in RNase-free water (Sigma, MO) and subsequently treated to remove genomic DNA using the Amplification Grade DNase I Kit (Sigma, MO). cDNA first-strand and second-strand cDNA were synthesized using the Superscript III First-Strand Synthesis SuperMix (Invitrogen, CA) and the Second-strand cDNA Synthesis Kit (BeyoTime, Jiangsu, China), respectively, following instructions.

### DNA and cDNA Library Construction and Sequencing

DNA and cDNA library preparation was conducted following the manufacturer’s instruction (Illumina). Briefly, DNA and cDNA fragmentation were carried out by Covaris S2 (Covaris, 01801-1721). The DNA and cDNA fragments were processed by end reparation, A-tailing, adapter ligation, DNA/cDNA size-selection, PCR reaction and products purification according to manufacturer’s instructions. A ∼200 bp DNA fragment sequences library and a ∼180 bp cDNA fragment sequences library were constructed for further sequencing. The base-calling pipeline (version Illumina Pipeline-0.3) was used to process the raw fluorescence images and call sequences. Raw reads with >10% unknown nucleotides or with >50% low quality nucleotides (quality value <20) were discarded [9]. Three sequencing depths, i.e. 0.5 Gbp, 1.0 Gbp, and 2.4 Gbp reads, were applied for both metagenomic and metatranscriptomic datasets.

### Bioinformatic Analyses

#### Combined taxonomic domain information analysis

Combined taxonomic domain information analysis was conducted at the MG-RAST (Meta Genome Rapid Annotation using Subsystem Technology, v3.1) server at the Argonne National Library (http://metagenomics.nmpdr.org), which provides several methods to access the different data types, including phylogenetic and metabolic reconstructions, and has the ability to compare the metabolism and annotations of one or more metagenomes [8], [26]. MG-RAST also provides protein similarities analysis for sequences pasted filtration, including both function classification and function annotation. The protein similarity was carried out using BLAT against M5NR protein database (http://metagenomics.nmpdr.org), which is an integration of many sequence databases into a single and searchable database. A single similarity search at this server will allow retrieving similarities to several databases, including NCBI-nr, KEGG, SEED, and etc. In the present study, ∼2.4 Gbp DNA dataset (MG-RAST ID: 4467420.3 and 4467390.3) and ∼2.4 Gbp RNA dataset (MG-RAST ID: 446943.3 and 4466567.3) were used for most of the analysis.

#### Taxonomic classification

Taxonomic classification was conducted by BLASTN against SILVA SSUref and LSUref databases release 108 with an e-value of 1e^−6^ [8] first, respectively, and then followed by annotation of BLAST output files using MEGAN [27]. This was performed by the lowest common ancestor algorithm that assigned rDNA or rRNA sequences to the lowest common ancestor in the taxonomy from a subset of the best scoring matches in the BLAST result (absolute cutoff: BLAST bitscore 86, relative cutoff: 10% of the top hit) [8] using MEGAN according to these cutoffs to select hit reads for annotation. SSU rRNA sequences were selected to carry out taxonomic analysis.

#### Global gene expression classification

To get whole metabolic pathways information, global gene expression was annotated with SEED Subsystems in MG-RAST with the total 2.4 Gbp DNA and 2.4 Gbp cDNA datasets at a cutoff of e-value <1e^−5^ [28], and visualized using KEGG mapper (an internal tool based on the KEGG pathway mapping system).

To evaluate the influence of different sequencing depths on pathways annotations, four sub-datasets (DNA: 0.5, and 1.0 Gbp, cDNA: 0.5 and 1.0 Gbp) were generated randomly generated from the total 2.4 Gbp DNA and 2.4 Gbp cDNA datasets and applied for annotation against SEED Subsystems at e-value <1e^−5^ [28].

#### Nitrogen metabolic pathway analysis

To study further on nitrogen metabolism, especially nitrification, denitrification, ammonification, and nitrogen fixation processes, which closely related to nitrogen removal in the AS system, Level 2 SEED subsystems of MG-RAST was applied to annotate nitrogen metabolism related genes. Additionally the nitrogen removal related sequences identified based on MG-RAST BLAT results were extracted and applied to BLASTX against NCBI-nr database at an e-value of 1e^−5^ [29]. The BLAST results were visualized with MEGAN KEGG analyzer [27] at a threshold of bitscore >50.

Further analysis was conducted to count the hit numbers of the sequences of corresponding enzymes subunits in nitrogen metabolism and the ratio of cDNA/DNA hit reads numbers of these enzymes from MEGAN annotation data. The present results only displayed the subunits whose minimal hit numbers were >10 in either DNA or cDNA dataset.

#### Taxonomic analysis of the sources of five enzyme-coding gene sequences and mRNA sequences

MG-RAST BLAT results, which contained species information, were used to assign the ammonia monooxygenase, hydroxylamine oxygenase, hydroxylamine reductase, nitrous oxidase, and nitrogenase to specific bacteria at the genus level. In detail, the BLAT results from two 2.4 Gbp DNA and cDNA datasets were filtrated at thresholds of maximum e-value of 1e^−5^, minimum identity cutoff of 85% and minimum alignment length cutoff of 80%. Then enzymes coding sequences, including both DNA and cDNA sequences, were displayed in a heat map with genus level affiliations and gene abundance. Only those genera with more than two hits in cDNA dataset or more than 20 hits in DNA dataset were displayed in the heat map which was generated using MATLAB (version 7.12.0).

## Supporting Information

Figure S1 **Microbial community profile revealed by DNA and cDNA datasets.** DNA and cDNA datasets were BLASTed with SILVA SSUref database and assigned with MEGAN.
(TIF)

Figure S2 **Global functional analysis using KEGG mapper in MG-RAST.** Three colors represented the regions covered by DNA only (blue), cDNA only (red), and both (purple).
(TIF)

Figure S3 **RNA qualification was tested by electrophoresis.**
(TIF)

Table S1 **Routine parameters monitored in Stanley WWTP.**
(DOCX)
