# Correction: Metagenomic and Metatranscriptomic Analysis of Microbial Community Structure and Gene Expression of Activated Sludge

**DOI:** 10.1371/journal.pone.0243233

**Published:** 2020-11-30

**Authors:** Ke Yu, Tong Zhang

Data repository information is missing from the article. Please find repository information here: Data is available in Sequence Read Archive: https://www.ncbi.nlm.nih.gov/bioproject/PRJNA602123/.
