# Supplementary figures and images for: Metagenomic and Metatranscriptomic Analysis of Microbial Community Structure and Gene Expression of Activated Sludge
Source: PLoS One. 2012 May 30;7(5):e38183. doi: 10.1371/journal.pone.0038183 (PMC3364235; doi:10.1371/journal.pone.0038183)

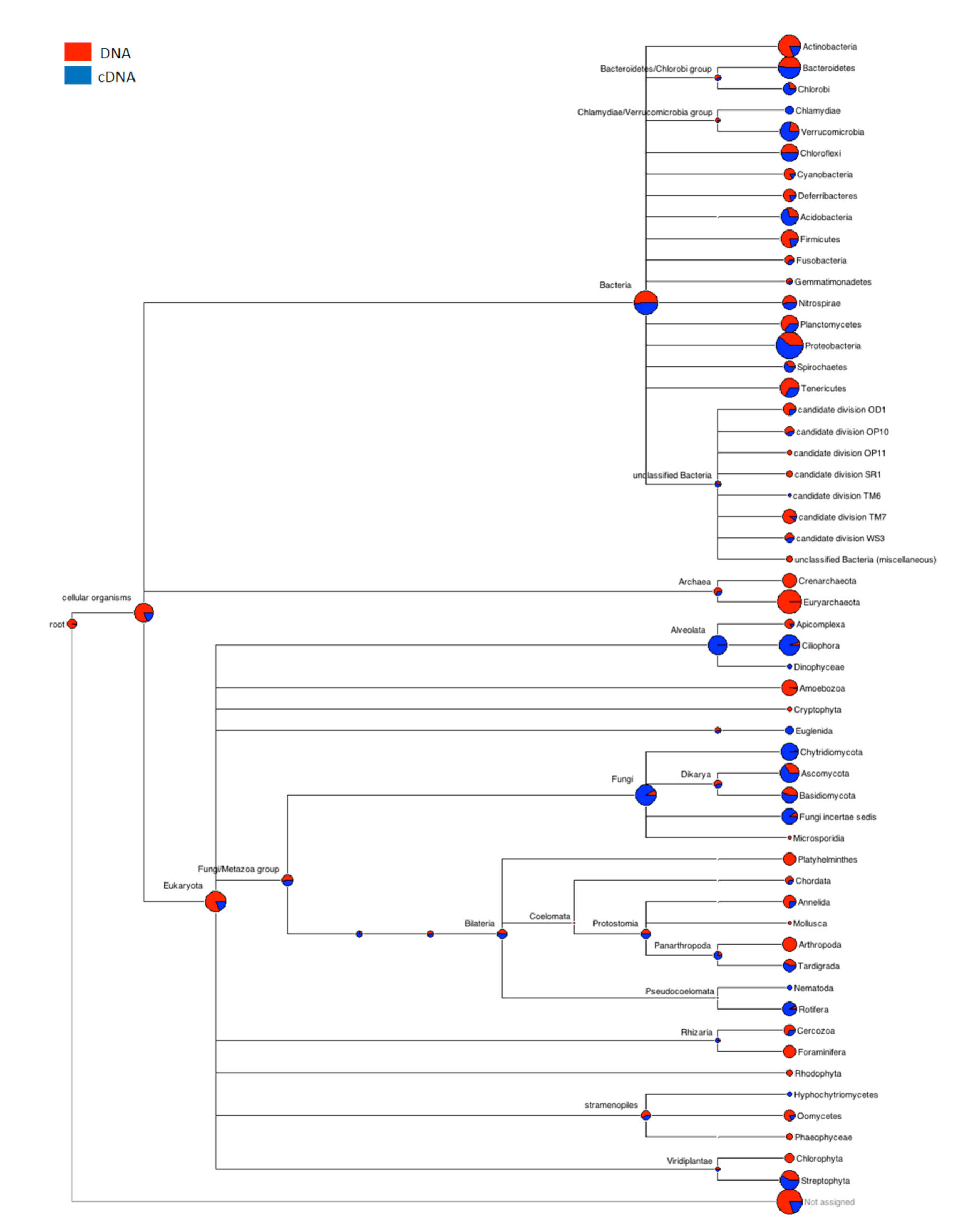

Supplement: Figure S1 — Microbial community profile revealed by DNA and cDNA datasets. DNA and cDNA datasets were BLASTed with SILVA SSUref database and assigned with MEGAN. (TIF) [file pone.0038183.s001.tif]

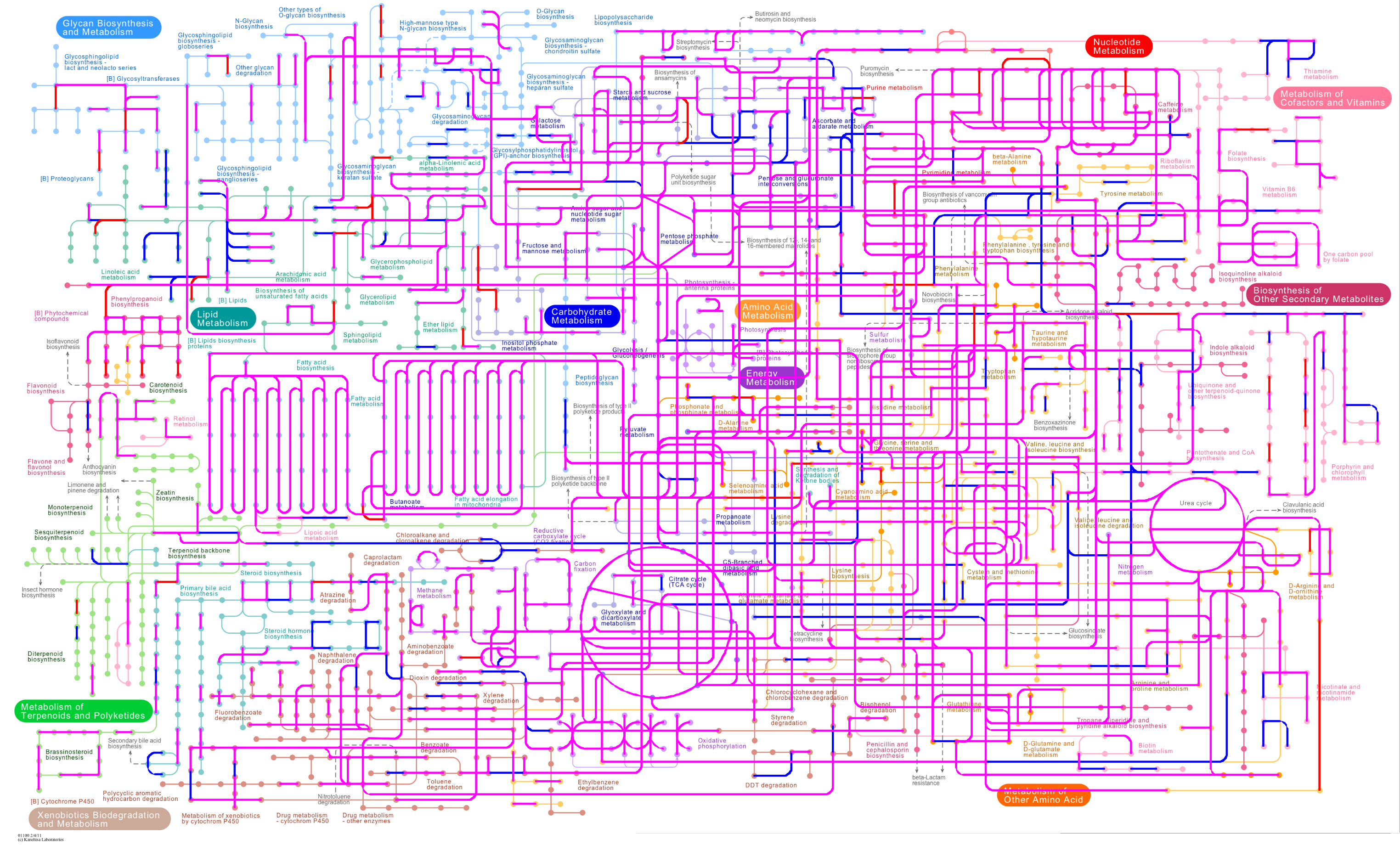

Supplement: Figure S2 — Global functional analysis using KEGG mapper in MG-RAST. Three colors represented the regions covered by DNA only (blue), cDNA only (red), and both (purple). (TIF) [file pone.0038183.s002.tif]

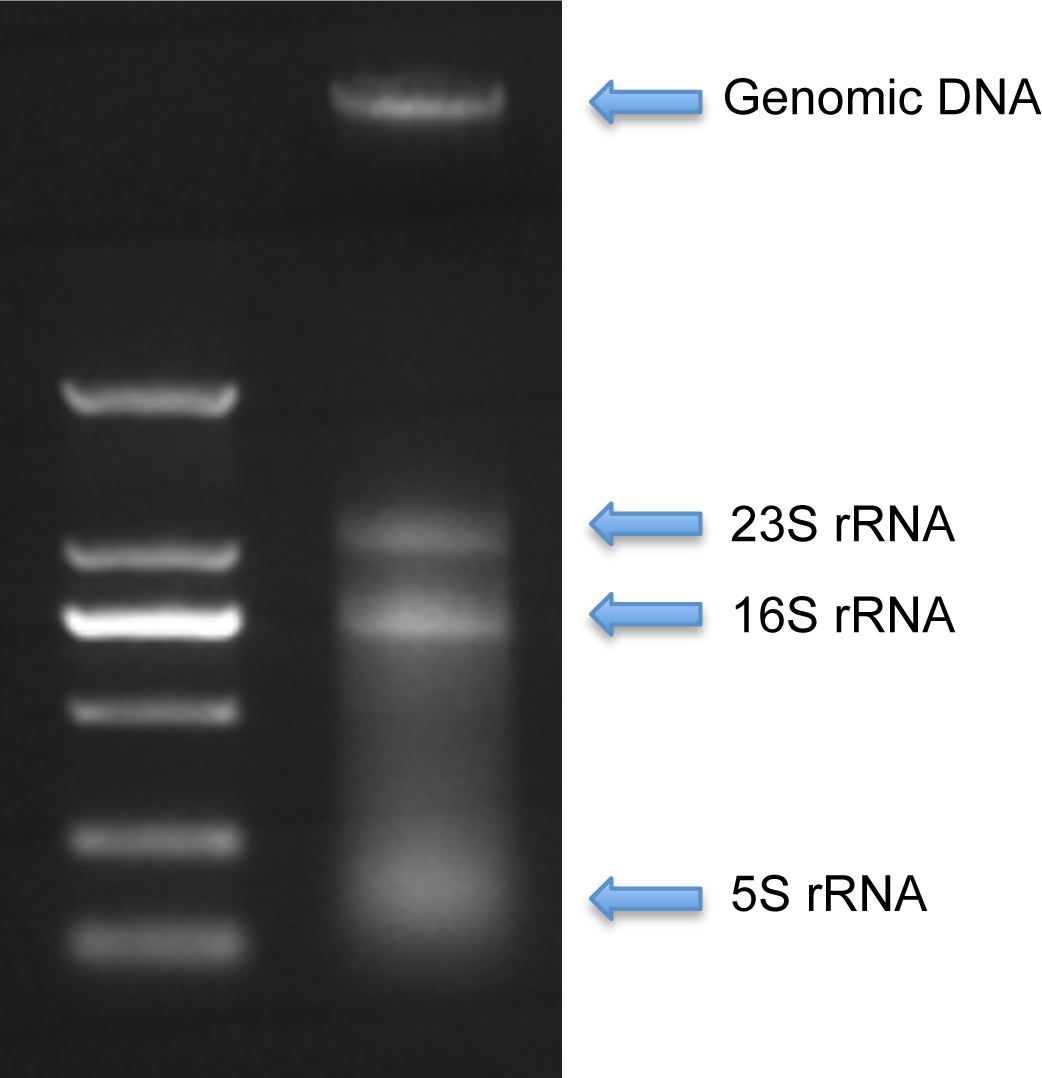

Supplement: Figure S3 — RNA qualification was tested by electrophoresis. (TIF) [file pone.0038183.s003.tif]
