# Supplementary material for: Metagenomic and Metatranscriptomic Analysis of Microbial Community Structure and Gene Expression of Activated Sludge
Source: PLoS One. 2012 May 30;7(5):e38183. doi: 10.1371/journal.pone.0038183 (PMC3364235; doi:10.1371/journal.pone.0038183)
Supplement: Table S1 — Routine parameters monitored in Stanley WWTP. (DOCX) [file pone.0038183.s004.docx]

Table S1. Routine parameters monitored in Stanley WWTP.

|  | Influent | | | | | Effluent | | | | |
| --- | --- | --- | --- | --- | --- | --- | --- | --- | --- | --- |
|  | pH | BOD (mg L^-1^) | COD (mg L^-1^) | NH_3_-N (mg L^-1^) | TP (mg L^-1^) | pH | BOD (mg L^-1^) | COD (mg L^-1^) | NH_3_-N (mg L^-1^) | TP (mg L^-1^) |
| Jan. 2010 | 6.7 | 594 | 1732 | 19 | 16 | 6.7 | <3 | 15 | 0.3 | 0.8 |
| Feb. 2010 | 6.6 | 379 | 1098 | 18 | - | 6.8 | <3 | - | 0.4 | - |
| Mar. 2010 | 6.8 | 448 | 1026 | 21 | 11 | 6.8 | <3 | 14 | 0.2 | 0.6 |
| Apr. 2010 | 6.8 | 435 | 1911 | 22 | 14 | 6.8 | <3 | 14 | 0.1 | 0.2 |
| May. 2010 | 6.9 | 271 | 586 | 20 | 8 | 6.9 | <3 | 10 | 0.1 | 0.6 |
| Jun. 2010 | 7 | 191 | 456 | 16 | 8 | 6.9 | <3 | 15 | 0.2 | 0.7 |
| Jul. 2010 | 7 | 162 | 341 | 14 | 3 | 7 | <3 | 12 | 0.2 | 0.9 |
| Aug. 2010 | 6.8 | 219 | 417 | 16 | 5 | 6.9 | <3 | 17 | 0.1 | 1.3 |
| Sep. 2010 | 7 | 170 | 344 | 16 | 5 | 6.8 | <3 | <10 | 0.2 | 0.8 |
| Oct. 2010 | 6.9 | 172 | 568 | 18 | 6 | 6.9 | <3 | 14 | 0.1 | 0.7 |
| Nov. 2010 | 7 | 166 | 498 | 16 | 7 | 6.8 | <3 | 12 | 0.2 | 0.9 |
| Dec. 2010 | 6.9 | 180 | 492 | 16 | 6 | 6.7 | <3 | 28 | 0.5 | 1.2 |
| Jan. 2011 | 7.1 | 209 | 413 | 18 | 4 | 6.7 | 4 | 17 | 0.6 | 1.1 |
| Feb. 2011 | 7.1 | 158 | 378 | 19 | 5 | 6.8 | 4 | 23 | 0.1 | 1.7 |
| Mar. 2011 | 7 | 167 | 554 | 15 | 5 | 6.8 | <3 | 15 | 0.2 | 1.6 |
| Apr. 2011 | 7 | 187 | 553 | 15 | 8 | 6.9 | 3 | 15 | 0.3 | 0.6 |
| May. 2011 | 6.8 | 171 | 564 | 21 | 8 | 7 | 8 | 16 | 0.3 | 1.2 |
| Jun. 2011 | 6.6 | 341 | 1170 | 18 | 7 | 6.8 | 8 | - | 0.2 | 1.1 |
| Jul. 2011 | 6.7 | 207 | 778 | 20 | 5 | 7 | 8 | 22 | 0.2 | 0.9 |
| Aug. 2011 | 6.8 | 150 | 405 | 21 | 6 | 7.2 | 7 | 38 | 0.2 | 1.4 |
| Sep. 2011 | 6.8 | 243 | 540 | 19 | 9 | 7 | 6 | 18 | 0.3 | 2.1 |
| Oct. 2011 | 6.7 | 205 | 466 | 17 | 5 | 7.2 | 6 | 16 | 0.2 | 1.8 |
| Nov. 2011 | 7 | 209 | 433 | 17 | 7 | 7.4 | 6 | 17 | 0.3 | 1.8 |
| Dec. 2011 | 6.9 | 179 | 530 | 18 | 7 | 7.2 | 6 | <10 | 0.2 | 1.2 |
| Average | 6.9 | 242.29 | 677.29 | 17.99 | 6.9 | 6.9 | 2.8 | 14.5 | 0.2 | 1.1 |
